# The prospect of artificial intelligence to personalize assisted reproductive technology

**DOI:** 10.1038/s41746-024-01006-x

**Published:** 2024-03-01

**Authors:** Simon Hanassab, Ali Abbara, Arthur C. Yeung, Margaritis Voliotis, Krasimira Tsaneva-Atanasova, Tom W. Kelsey, Geoffrey H. Trew, Scott M. Nelson, Thomas Heinis, Waljit S. Dhillo

**Affiliations:** 1 Department of Metabolism, Digestion, and Reproduction, Imperial College London, London, UK; 2 Department of Computing, Imperial College London, London, UK; 3 UKRI Centre for Doctoral Training in AI for Healthcare, Imperial College London, London, UK; 4 Imperial College Healthcare NHS Trust, London, UK; 5 Department of Mathematics and Statistics, University of Exeter, Exeter, UK; 6 Living Systems Institute, University of Exeter, Exeter, UK; 7 EPSRC Hub for Quantitative Modelling in Healthcare, University of Exeter, Exeter, UK; 8 School of Computer Science, University of St Andrews, St Andrews, UK; 9 The Fertility Partnership, Oxford, UK; 10 School of Medicine, University of Glasgow, Glasgow, UK; 11 Biomedical Research Centre, University of Bristol, Bristol, UK

**Keywords:** Machine learning, Endocrine reproductive disorders, Infertility, Embryology

## Abstract

Infertility affects 1-in-6 couples, with repeated intensive cycles of assisted reproductive technology (ART) required by many to achieve a desired live birth. In ART, typically, clinicians and laboratory staff consider patient characteristics, previous treatment responses, and ongoing monitoring to determine treatment decisions. However, the reproducibility, weighting, and interpretation of these characteristics are contentious, and highly operator-dependent, resulting in considerable reliance on clinical experience. Artificial intelligence (AI) is ideally suited to handle, process, and analyze large, dynamic, temporal datasets with multiple intermediary outcomes that are generated during an ART cycle. Here, we review how AI has demonstrated potential for optimization and personalization of key steps in a reproducible manner, including: drug selection and dosing, cycle monitoring, induction of oocyte maturation, and selection of the most competent gametes and embryos, to improve the overall efficacy and safety of ART.

## Introduction

**Fig. 1:**
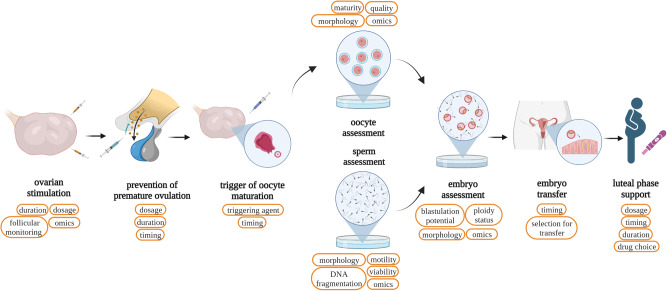
Potential targets for artificial intelligence in assisted reproductive technology. Potential targets for the application of artificial intelligence and machine learning methods during clinical and embryological steps in assisted reproductive technology (ART). Investigations of infertility and pre-treatment counseling are not captured here and discussed independently in Section Pre-treatment counseling. The order and timings of the steps can differ depending on the ART protocol used. Figure created with BioRender.com.

Since the birth of the first baby conceived through in vitro fertilization (IVF) in 1978, the development of assisted reproductive technology (ART) has evolved significantly. Over the last 40 years, ART has provided infertile couples with the possibility to conceive, culminating in the birth of over eight million children^1^. IVF protocols are complex and require intensive monitoring, with clinicians and embryologists responsible for several key decision points prior to and during the cycle (Fig. 1). Although several of these decisions have a solid evidence base, many are highly subjective and will vary immensely based on clinical experience with an inevitable non-reproducible impact on clinical outcomes—leading to the mantra that ART is an art.

Given these limitations, there is increasing recognition that alternative data-driven approaches that harness the large number of ART cycles undertaken and facilitate objective, consistent, and optimal decision-making may be associated with improved outcomes. Large amounts of data generated during IVF cycles have enabled interdisciplinary researchers to propose artificial intelligence (AI) methodologies to drive individualized approaches. These have ranged from algorithmic drug dosing tools, to ‘human-in-the-loop’ AI clinical decision support systems (CDSSs) for embryo selection, whereby humans are supported by AI but ultimately make the final decision. Harnessing the symbiosis between the experience of clinicians, and personalized recommendations from AI models based on the one million cycles undertaken annually, has the potential to synergistically improve clinical outcomes. In this review, we examine current implementations of AI models within ART, and future prospects concerning their utility, efficacy, and application in the field.

## Artificial intelligence methods for assisted reproductive technology

**Fig. 2:**
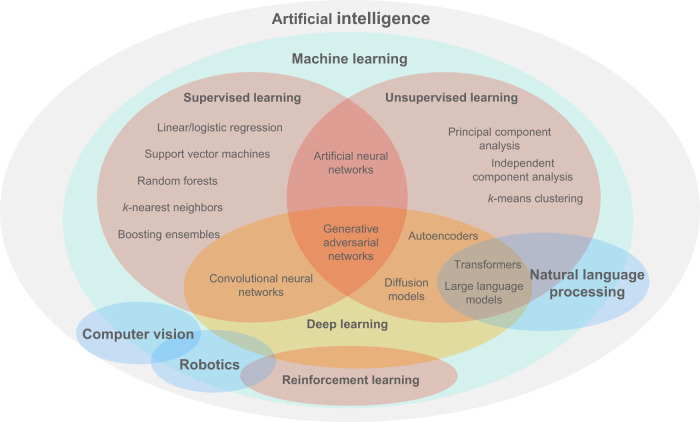
The artificial intelligence landscape. A Venn diagram providing a holistic view of the artificial intelligence (AI) landscape, with a particular focus on machine learning (ML) methods. ML is a subfield that is often used in conjunction with other AI subfields, such as computer vision. Some methods can be used in alternative learning frameworks however their most common current manifestations are presented here.

AI is an overarching term that encompasses a growing number of subfields including machine learning (ML), robotics, and computer vision (Fig. 2). Principally, ML methods can learn patterns from data and draw inferences, and therefore build models that optimize/personalize ART protocols for a specified outcome. Traditionally, ML can be under either a supervised, unsupervised, or reinforcement learning framework. In supervised learning, data are labeled as inputs and outputs with the goal being to develop models that capture the relationship between the two, which can be used to predict outputs when presented with new, unseen inputs. Conversely, in unsupervised learning, models are built to capture the structure (e.g., clustering) of data with no output labels (‘unlabeled’) that can be used to interpret new, or generate synthetic data. Reinforcement learning trains an ML agent that interacts with a defined environment towards achieving a goal and receives a ‘reward’ for its actions.

Supervised methods include decision trees, linear/logistic regression, *k*-nearest neighbors, support vector machines, random forests, artificial neural networks (ANNs), and more. Decision trees are models used to classify or predict outcomes based on input data. They can effectively capture non-linear relationships and can be visualized intuitively as tree-like structures: starting from the root, each branch represents a decision rule to select which subsequent branch should be followed; the final nodes (‘leaves’) of the tree represent outcomes. Extending this to an ‘ensemble’ of trees inspires the random forest algorithm, where each tree is trained on a random partition of the data and its input features. The final prediction is determined by a voting mechanism, combining the predictive power of all decision trees. This generally makes the model less prone to ‘overfitting’, a phenomenon whereby a model may perform very well on training data but poorly on new, unseen data. Supervised methods have widespread applications with tabular (i.e., numerical or categorical) outcomes in ART.

ANNs are networks of connected computational units representing artificial neurons—they receive inputs, process them, and signal the result to other neurons connected to them, in a multi-layered structure (e.g., the multi-layer perceptron algorithm). The input layer receives data to be processed, and the output layer presents the model output. The strengths (‘weights’) of connections between artificial neurons comprise the parameters of the ANN and are calibrated during model training.‘Deep’ learning is ANNs with complex architectures comprising many layers, an example being convolutional neural networks (CNNs), useful for spatial, grid-like data (e.g., embryo images).

As for unsupervised methods, *k*-means is a popular algorithm for clustering data into *k*-groups based on the distance of data points from the centroid of each group. Another example is generative adversarial networks (GANs), where one network is trained to generate synthetic data whilst the other discriminates synthetic from real data. The two networks are trained in parallel, competing as adversaries, resulting in better discrimination between synthetic and real data. Multimodal generative AI has recently caught mass media attention, especially through both text (e.g., ChatGPT and Med-PaLM) and text-to-image generators (e.g., DALL-E), which have been evolving rapidly in performance since their inception^2^. These frameworks bring together large language models (LLMs), a type of natural language processing built with ANNs, and diffusion models, an alternative generative methodology to GANs based on iterative de-noising to estimate how image data are distributed to therefore generate a desired image^3^.

During model development, it is standard practice to use ‘training’, ‘validation’, and ‘test’ datasets: ‘training’ to fit the model, ‘validation’ to fine-tune the model’s hyperparameters, and a ‘test’ set to independently evaluate the model’s performance. For generally more reliable estimates of model performance, cross-validation can be used to evaluate the model on multiple training/validation data splits. Using test datasets that are externally unseen and temporally different (e.g., from a different clinic) can provide further reassurance of a model’s generalizability. The fundamental choice of ML algorithm for a certain task is multifaceted and often driven by contextual reasoning. Nevertheless, Table 1 presents some rules-of-thumb regarding popular ML methods (Fig. 2) within the context of ART.

**Table 1:** Rules-of-thumb for most suitable machine learning algorithms

AI method	Learning type	Common tasks	Must suitable data types	Quantity of data required	Interpretability	Example use in ART?
Linear/Logistic regression	Supervised	C&R	Numerical	**++**	**+++**	Optimizing trigger day timing^27^
Decision tree	Supervised	C&R	Numerical, categorical	**++**	**+++**	Decision-making during OS^30^
*k*-NN	Supervised	C&R	Numerical, categorical	**+**	**++**	Optimizing starting dose during OS^11^
SVM	Supervised	C&R	Numerical, categorical	**++**	**++**	Streamlining monitoring of patients during OS^31^
Random forest	Supervised	C&R	Numerical, categorical	**++**	**++**	Predicting risk of OHSS during OS^32^
CNN	Supervised, unsupervised	C&R, clustering	Image, audio, text	**+++**	**+**	Predicting ploidy status of an embryo^100^
*k*-means	Unsupervised	Clustering	Numerical	**++**	**++**	Effect of sperm parameters on IVF outcomes^64^
GAN	Unsupervised	Generative	Image, time-series, text	**+++**	**+**	Generating synthetic embryo images^73^
LLM	Unsupervised	Generative	Text	**+++**	**+**	Pre-treatment counseling^6^

Rules-of-thumb in determining the most suitable machine learning algorithm for a task with relevant examples of their application. Three plus signs imply the highest requirement or capacity, and one plus sign the lowest. For example, the convolutional neural network (CNN) supports several data types, and generally requires high quantities of data (i.e., thousands) for adequate performance, but exhibits poor interpretability (i.e., ‘black-box’). Conversely, *k*-nearest neighbors (*k*-NN) can work well even with only hundreds of data samples, and the weighting of predictors can be reasonably estimated for interpretability purposes. *AI* artificial intelligence, *ART* assisted reproductive technology, *C&R* classification and regression, *SVM* support vector machine, *CNN* convolutional neural network, *GAN* generative adversarial network, *LLM* large language model, *OS* ovarian stimulation, *OHSS* ovarian hyperstimulation syndrome.

## Pre-treatment counseling

Classically, age-stratified population estimates have been used to inform patients of their overall chance of success, however, these often fail to incorporate important determinants of outcome such as previous treatment cycle attempts or for treatment-naive patients their ovarian reserve and likely ovarian response. To try to tailor these models further both population data and clinic-specific datasets have been used to develop models for a variety of outcomes including for cumulative live births across multiple cycles^4^. These models are now being used by both patients and a range of stakeholders to manage access to care (national healthcare services, insurance providers) and clinics, or third-party companies offering shared-risk financial programs^5^. Moreover, the emergence of AI chatbots using LLMs could improve efficiency in the initial assessment of infertility. A recent ‘Fast Track to Fertility’ program using semi-automated two-way text messages reduced the time to complete a workup by 50%^6^. The deployment of LLMs for fertility assessment offers unique challenges and currently remains experimental, whilst the frameworks for validation and regulation of such systems are yet to be formalized^2,7^.

## Gonadotropin dosing for ovarian stimulation

Ovarian stimulation (OS) is used to stimulate the growth of multiple ovarian follicles in order to result in multiple oocytes for retrieval^8^. IVF treatment is a profligate process as not all follicles yield oocytes, not all oocytes will fertilize, and not all embryos will develop, implant, or be capable of becoming healthy babies. Various preparations of gonadotropins exist but most will contain a supra-physiological amount of follicle-stimulating hormone (FSH) to extend the ‘FSH-window’ by maintaining high FSH levels, and induce multi-follicular growth^9^. Optimization of the gonadotropin dosing regimen can maximize the number of follicles with respect to ovarian potential^9^. As such, an optimal initial dose of FSH can ensure sufficient follicles are recruited, whilst avoiding the recruitment of too many follicles (often defined as >15 oocytes at pickup), and an increased risk of ovarian hyperstimulation syndrome (OHSS)^8,10^.

The application of ML approaches to retrospective datasets for model learning has demonstrated the potential to personalize FSH dose as summarized in Table 2. Fanton et al. aimed to identify the 100 most similar patient profiles to each patient, to then generate individualized dose-response curves^11^. The authors reported limitations including a protocol-agnostic approach, and that 87% of cycles included both pure FSH and Menopur (for luteinizing hormone (LH)-like activity) during OS^11^. The methodology was further evaluated against the national US database (SART CORS) including 365,473 patients and reported upon in conference proceedings^12^. The results similarly predicted that an increased number of two-pronuclear fertilized embryos (2PNs) and blastocysts could be retrieved whilst using significantly lower total FSH doses, key in reducing high medication costs for patients^12^. Nevertheless, OS protocols vary across clinical practice, and the generated dose-response curves presented less confidence in predicting oocytes with lower doses of FSH administration^11^, which are the norm in Europe (where 150-225 IU is suggested for normal responders^8^). Therefore, it is necessary to determine whether the proposed models are directed at certain geographies or intend to be universal. Setting a precedent for the conduct of future multi-center studies is central to achieving either objective—Ferrand et al. successfully leveraged a federated learning framework^13^, a potentially effective approach that allows data to be kept decentralized and private, whilst deploying ML models for collaborative training between clinics^14,15^.

**Table 2:** Ovarian stimulation assessment studies using artificial intelligence

Study	Aims of study	Outcomes of interest	Dataset	AI methods	Results
Andersen et al. (2017)^22^	-Assess the efficacy of individualized dosing of follitropin delta (r-FSH) by body weight and AMH vs. conventional follitropin alpha dosing.	-Ongoing pregnancy and implantation rates. -Patient safety and level of OHSS.	-Randomized, multi-center, assessor-blinded, noninferiority trial across 11 countries with 1329 women aged 18–40 years. -Cycles were under a fixed day-6 GnRH-ant protocol with a GnRH-a or hCG trigger for oocyte maturation.	Proprietary algorithm	-Ongoing pregnancy (30.7% vs. 31.6%) and implantation rates (29.8% vs. 30.7%) were similar. -2.3% of patients required measures against OHSS, compared to 4.5% in conventional dosing. -A similar efficacy and improved safety was observed, with significantly less FSH used.
Letterie and Mac Donald (2020)^30^	-CDSS to conduct the day-to-day management of OS.	Acc., TPR, and PPV of the algorithm to support critical decision-points: (1) stop or continue stimulation? (2) if stop, trigger or cancel the cycle? (3) if continue, after how many days to follow-up? (4) any FSH dose adjustments?	-Retrospective dataset with 3,159 IVF cycles from a single center. 556 cycles (17.6%) held out for independent testing of the CDSS vs. 12 clinicians. -Cycles were either the flexible GnRH-ant protocol or MDL, with an hCG trigger for oocyte maturation. Input variables: E2 levels, follicle sizes during scans, cycle day during OS, FSH dose during OS.	Evaluated 5 model types: Decision trees, random forest, support vector machine, logistic regression, ANN.	(1) Acc. 0.92: continue (TPR 0.94; PPV 0.95), stop (TPR 0.85; PPV 0.85). (2) 0.96: trigger (0.98; 0.97), cancel (0.75; 0.78). (3) 0.87: 1 day (0.89; 0.86), 2 days (0.84; 0.88), 3 days (0.91; 0.86). (4) 0.82: same (0.96; 0.84), decrease (0.23; 0.67), increase (0.25; 0.55). -A seminal proof-of-concept for a CDSS during OS which generally agreed with evidence-based decisions.
Qiao et al. (2021)^24^	-Assess the efficacy of individualized dosing of follitropin delta (r-FSH) by body weight and AMH vs. conventional follitropin alpha dosing in Asian patients.	-Ongoing pregnancy rate and LBR. -Patient safety and incidence of OHSS.	-Randomized, multi-center, assessor-blinded, noninferiority trial across China, South Korea, Vietnam, and Taiwan, with 1009 women aged 20–40 years -Cycles were under a fixed day-6 GnRH-ant protocol with a GnRH-a or hCG trigger for oocyte maturation.	Proprietary algorithm	-Ongoing pregnancy (31.3% vs. 25.7%) was similar between individualized and conventional dosing. -LBR was significantly higher in individualized dosing (31.3% vs. 24.7%). -Incidence of early OHSS was significantly lower (5.0% vs. 9.6%). -A similar efficacy and improved safety was observed, with significantly less FSH used.
Ishihara et al. (2021)^23^	-Assess the efficacy of individualized dosing of follitropin delta (r-FSH) by body weight and AMH vs. conventional follitropin beta dosing in Japanese patients.	-Number of oocytes retrieved and LBR. -Patient safety and incidence of OHSS.	-Randomized, multi-center, assessor-blinded, noninferiority trial in Japan with 347 women aged 20–40 years.	Proprietary algorithm	-Noninferiority shown in the number of oocytes retrieved with individualized dosing (9.3 vs. 10.5). -LBR was 23.5% with individualized dosing vs. 18.6%. -Occurrence of OHSS was significantly less with individualized dosing (11.2% vs. 19.8%). -A similar efficacy and improved safety was observed, with significantly less FSH used.
Fanton et al. (2022)^11^	-Optimize starting OS dose with respect to maximizing the number of MIIs and usable blastocysts.	(1) Number of MIIs retrieved (MAE). (2) Expected benefits in reduced total OS dose requirements, and increased number of MIIs, 2PNs, and usable blastocysts, when comparing to propensity-matched patients under an optimal dosing strategy.	-Retrospective dataset with 18,591 cycles from three centers (1229, 11,233, 6129 cycles respectively). -87% of cycles had OS including Menopur; 13% had only pure FSH administered. -Input variables: age, BMI, baseline AMH and AFC.	*k*-NN regression (*k* = 100) using 5-fold CV. Logistic regression for propensity matching.	(1) MAE of 3.79 MIIs with *r*^2^ = 0.45. (2) 30% of cycles were dose-responsive and 64% flat-responsive. -Dose-responsive patients with an optimal dosing strategy were predicted to have 1.5 more MIIs, 1.2 2PNs, and 0.6 usable blastocysts, with 195 IU less total FSH. -Flat-responsive patients were predicted to have 0.3 more MIIs, 0.3 2PNs, and 0.2 usable blastocysts, with 1375 IU less total FSH. -Demonstrates potential for individualized OS with significantly reduced dosing requirements.
Correa et al. (2022)^16^	-Optimize starting OS dose with respect to the number of MIIs.	-Mean performance score in comparison to a clinician’s dosing strategy. The performance range was defined from -1 (dose too low) to +1 (dose too high), and 0 being ideal.	-Retrospective dataset of first cycles from 5 centers were analyzed. 2713 patients with a mean age of 37.7 ± 4.6 years, and a further 774 patients with a mean age of 38.3 ± 4.4 years held out for independent testing. -Input variables: age, BMI, AFC, AMH, and proven fertility (Yes/No).	Linear regression with 5-fold CV	-Algorithm aimed to optimize dosing strategy to achieve 12 MIIs. -Demonstrated potential to surpass the performance of standard practice. -Mean performance score in the test set was 0.89 (95% CI 0.88–0.90) versus the clinicians’ suggestions 0.84 (95% CI 0.82–0.86).
Xu et al. (2023)^17^	-Predicting (A) starting and (B) adjustment of OS dose with respect to the number of oocytes retrieved. -Developing an online tool for clinicians to use.	-Generalized *R*^2^ and RMSE of models A and B. -Development of a practical online tool for clinicians.	-Retrospective dataset with 621 antagonist cycles from a single center. 30% held out for independent testing. -Input variables for (A): AMH, AFC, basal FSH, age. -Input variables for (B): AMH, AFC, age, change in inhibin B.	Lasso regression	-Model (A) had *R*^2^ = 0.923 and RMSE = 0.224 in the test set. AMH contributed the most. -Model (B) had *R*^2^ = 0.909 and RMSE = 0.231 in the test set. Change in inhibin B contributed the most. -A practical online tool (‘POvaStim’) was successfully developed incorporating both models, which now awaits RCT validation.
Zieliński et al. (2023)^18^	-Predict the number of MIIs retrieved using both clinical and genetic features.	-RMSE (primary metric), MAE, and MAPE of models solely based on clinical data (A) and when augmented with genetic data (B).	-Retrospective dataset across 9 clinics from 6,043 patients, who had 9,090 IVF treatment cycles. -264 of the patients had genetic data available (with 516 IVF cycles). -Clinical and genetic features were iteratively added to reduce the RMSE of the models.	-Light Gradient Boosting Machine with 5-fold CV and ‘SHAP’ predictor analysis. -Genetic features were generated using classical bioinformatics analyses (e.g., haplotype construction).	-(A) RMSE = 3.53, MAE = 2.58, MAPE = 2.71. The final included features were AMH, AFC, age, number of cumulus-denuded oocytes and MIIs in the previous cycle attempt, and PCOS diagnosis. AMH was the most important predictor. -(B) RMSE = 3.35, MAE = 2.48, MAPE = 0.68. The final included features were the same as (A) in addition to IV8-6, IV41-8, and IV22-2 genetic features. AMH remained the most important predictor and was correlated to IV8-6. Haplotypes IV41-8 and IV22-2 both contributed to increasing the number of MIIs retrieved. -Seminal contribution to the capability of genetic data to augment the performance of clinical predictor models.
Ferrand et al. (2023)^13^	-Predict the number of oocytes retrieved from OS without transferring sensitive data.	(1) Number of oocytes retrieved (MAE) and MAPE. (2) Range of oocyte number, determined by 2 clinicians: (A) {0, 1–3, 4-7, 8-12, 13-20, 21-29, 30+} oocytes (B) {0, 1–5, 6-10, 11-18, 19-25, 25+} oocytes	-Retrospective dataset with 11,286 cycles from a single center. 20% of cycles held out for independent testing. -16 input variables considered: age, AMH, BMI, initial OS dose, basal E2, AFC, basal FSH, basal LH, infertility types, number of previous pregnancies, number of oocytes retrieved, protocol, OS drug type, WHO ovulatory disorder status, smoking status, basal testosterone, basal thyroid stimulating hormone.	-Light Gradient Boosting Machine and 5-fold CV. -‘SHAP’ predictor analysis was used.	(1) MAE = 4.21 oocytes; MAPE = 0.52. (2) A: MAE 0.73 bins of deviation. B: MAE 0.62 bins of deviation. -Overall 5 most important features across models: AFC, AMH, basal FSH, initial OS dose, and number of previous pregnancies. -Presents the feasibility of using federated learning to develop an oocyte prediction model.

Studies which use machine learning (ML) techniques to optimize gonadotropin dosing and duration during OS. *IVF* in vitro fertilization, *CDSS* clinical decision support system, *OS* ovarian stimulation, *Acc.* accuracy, *TPR* true positive rate (sensitivity), *PPV* positive predicted value, *FSH* follicle-stimulating hormone, *r-FSH* recombinant FSH, *GnRH-ant* gonadotropin-releasing hormone antagonist, *MDL* microdose leuprolide (flare), *GnRH-a* gonadotropin-releasing hormone agonist, *hCG* human chorionic gonadotropin, *E2* estradiol, *P4* progesterone, *AFC* antral follicle count, *AMH* anti-Müllerian hormone, *LH* luteinizing hormone, *ANN* artificial neural network, *MAE* mean absolute error, *R*^2^ coefficient of determination, *RMSE* root-mean-squared error, *MAPE* mean absolute percentage error, *PCOS* polycystic ovary syndrome, *CV* cross-validation, *BMI* body-mass index, *IU* international units, *MIIs* metaphase-II oocytes, *2PNs* two-pronuclear embryos, *k-NN* *k*-nearest neighbor, *LBR* live birth rate, *cLBR* cumulative LBR.

Recent studies have also focused on the effects of demographic, endocrine, and genetic data to optimize OS, and therewith predict the retrieval of mature oocytes^16–18^. Although these are retrospective studies, they highlight the need to explore available characteristics and further assess their impact on clinical outcomes when determining dosing regimens, whereby endocrine monitoring or genomic sequencing for ART cycles may be efficacious for some patients^19,20^. To best identify such predictors in an unbiased manner, the treatment cycles of patients should not exist in both the training and test sets^21^. An independent test set of patients should be partitioned at random, or if cross-validation is employed, cycles from the same patient must not exist across the training and test folds.

Ultimately, determining the efficacy of introducing individualized gonadotropin dosing algorithms into the clinic will require appropriate validation across different geographies. The three prospective international multi-center randomized controlled trials (RCTs) for follitropin delta (recombinant-FSH; Ferring Pharmaceuticals) that assess a unique algorithm to facilitate individualization of dose based on anti-Müllerian hormone (AMH) and body weight are an apt example of that critical step^22–24^. The retrospective studies in Table 2 would benefit from similar prospective validation in multiple centers to establish whether their adoption in the clinic is appropriate and of value for patients.

## Induction of oocyte maturation

Once multiple follicles have grown during OS, a hormonal trigger is administered to mature oocytes in preparation for retrieval. The triggering agent is most efficacious when follicles are neither too large nor too small^10^. In turn, AI/ML techniques have been harnessed to optimize the trigger day (TD) as summarized in Table 3. Our research team previously developed a random forest model to determine follicle sizes on TD that most contributed to the number of mature oocytes retrieved^25^. Maximizing the number of follicles sized 12-19 mm on TD was determined as optimal for yielding mature oocytes and could be used as a feature in conjunction with baseline endocrine characteristics to predict oocyte yield^19^.

**Table 3:** Trigger day assessment studies using artificial intelligence

Study	Aims of study	Outcomes of interest	Dataset	AI methods	Results
Abbara et al. (2018) ^25^	Follicle sizes on the day of trigger most likely to yield mature oocytes.	Optimal follicle sizes on TD.	Retrospective dataset with 499 patients. GnRH-ant protocol with hCG (*n* = 161); GnRH-a (*n* = 165); KP-54 (*n* = 173) triggers. Input variables: individual follicle diameters (in mm) from ultrasound scan on TD.	Random forest with 5-fold CV.	Follicles of diameter 12–19 mm were most contributory to the models following all three trigger types.
Abbara et al. (2020)^19^	Examine the relationship between endocrine changes following the use of different oocyte maturation triggers. Assess the relative importance of endocrine predictors when predicting mature oocyte yield.	(1) Accuracy in predicting the number of mature oocytes retrieved. (2) Relative importance of LH/hCG as an input variable.	Retrospective dataset with 499 patients. GnRH-ant protocol with hCG (*n* = 161); GnRH-a (*n* = 165); KP-54 (*n* = 173) triggers. Input variables: baseline endocrine characteristics, number of follicles sized 12–19 mm.	Performance comparison between a random forest with 5-fold CV and an ANN model.	Random forest had 88% accuracy within a tolerance level of 3 mature oocytes. The performance dropped to 83% when data on baseline LH/hCG levels were excluded. -The ANN had 57% accuracy.
Robertson et al. (2021)^32^	Finding the optimal tracking strategy for OS to minimize face-to-face interactions.	Earliest day during OS which can predict both the optimal TD and risk of OHSS accurately.	Retrospective dataset with 2128 cycles of 1731 women in a single center. 88.8% were GnRH-ant (fixed) cycles. An hCG trigger was used. Input variables: age, AFC, follicle count by size on each scan.	Random forest regressor for TD. Binary random forest classifier for OHSS prediction.	Day-5 was the earliest cycle day for predicting both outcomes accurately. The day-5 model had a MSE of 2.16 ± 0.12 for TD and AUC of 0.91 ± 0.01 for OHSS classification.
Hariton et al. (2021)^26^	Optimize TD timing to maximize 2PN and usable blastocyst yield.	Average improvement of 2PNs (primary outcome), and usable blastocysts vs. a clinician’s decision.	Retrospective dataset with 7,866 ICSI cycles. 1,967 cycles (25%) held out for independent testing. GnRH-ant, LD21, Lupron stop, flare, or mini-IVF (natural cycle) protocols were used. Input variables: age, BMI, number of follicles of {6-10, 11-15, 16-20, 21-25} mm, E2 level, protocol type, TD.	Light Gradient Boosting Machine with bagging.	Average improvement: 3.015 more 2PNs (95% CI 2.626, 3.371) and 1.515 more usable blastocysts (95% CI 1.134,1.871). Given physician agreement with the model (52.57% for 2PNs, 61.89% for blastocysts): 1.430 more 2PNs, and 0.577 more usable blastocysts. Follicle sizes 16-20mm were most contributory to the model performance.
Letterie et al. (2022)^31^	Workflow optimization of OS: (1) single ‘best day’ for monitoring during OS; (2) predict optimal TD; (3) predict total number of retrieved oocytes.	Acc., TPR, and PPV of total number of retrieved oocytes and mature oocytes stratified into: 0–10, and >10. MAE of determining the aims of (1) and (3).	Retrospective data-set with 1591 IVF cycles from a single center. 318 cycles (20%) held out for independent testing. An hCG or Lupron trigger was used. Pre-cycle selected input variables: age, AMH. ‘Best day’ selected input variables: E2 levels, follicle counts and sizes, day of cycle during OS, dose of FSH during OS.	Stacking ensemble model comprising: linear regression, random forest, extra trees regression, *k*-nearest neighbors, XGBoost.	(1) ‘Best day’ prediction: MAE 1.355. (2) Variance of 0-3 days for trigger choice showed “little impact” to oocytes retrieved. (3) Total number of oocytes: MAE 3.517. Total retrieved oocytes: Acc. 0.77; 0-10 oocytes (TPR 0.80; PPV 0.79); >10 oocytes (TPR 0.74; PPV 0.74). Total retrieved mature oocytes: Acc. 0.89; 0-10 oocytes (0.91; 0.89); >10 oocytes (0.86; 0.88). Total number of oocytes: MAE 3.517.
Fanton et al. (2022)^27^	Optimize TD timing to maximize MIIs, 2PNs, and blastocyst yield.	Average number of MIIs (primary outcome), 2PNs, and usable blastocysts.	Retrospective dataset with 30,278 cycles from 3 centers (2555, 3051, 14,672 cycles). 20% held out for independent testing. No available protocols were excluded. Pre-cycle input variables: age, BMI, AFC, previous IVF cycles, AMH, E2 level, cycle length (in days). Mid-cycle input variables: number of follicle scans during OS, E2 levels during OS, number of follicles of size {<11, 11–13, 14–15, 16–17, 18–19, >19} mm on TD.	Multivariable linear regression	Patients with early triggers had 2.3 fewer MIIs, 1.8 fewer 2PNs, and 1.0 fewer usable blastocysts when compared to propensity-matched on-time triggers. Patients with late triggers had 2.7 fewer MIIs, 2.0 fewer 2PNs, and 0.7 fewer usable blastocysts when compared to propensity-matched on-time triggers. Only follicle sizes and E2 were used in the final model.

Studies that use machine learning (ML) techniques to optimize trigger day timing during OS. *IVF* in vitro fertilization, *CDSS* clinical decision support system, *OS* ovarian stimulation, *TD* trigger day, *Acc.* accuracy, *TPR* true positive rate (sensitivity), *PPV* positive predicted value, *FSH* follicle-stimulating hormone, *GnRH-a* gonadotropin-releasing hormone agonist, *GnRH-ant* gonadotropin-releasing hormone antagonist, *hCG* human chorionic gonadotropin, *KP-54* kisspeptin-54, *E2* estradiol, *P4* progesterone, *AFC* antral follicle count, *AMH* anti-Müllerian hormone, *LH* luteinizing hormone, *LD21* long day 21, *ANN* artificial neural network, *MAE* mean absolute error, *MSE* mean squared error, *AUC* area under curve, *CV* cross-validation, *BMI* body-mass index, *IU* international units, *MIIs* metaphase-II oocytes, *2PN* two-pronuclear embryo, *k-NN* *k*-nearest neighbor, *cLBR* cumulative live birth rate.

A more recent study leveraged patients that had ultrasound scans both on the day before trigger, and on the true TD, to learn why a clinician might decide to wait a further day to trigger^26^. They found follicles sized 16-20 mm as most contributory in determining optimal TD, and predicted superior outcomes in terms of 2PN and blastocyst yield compared solely to a clinician’s decision^26^. With a similar methodology but using a simpler model, Fanton et al. confirmed the findings with even further granularity and showed follicles sized 14-15 mm were most predictive on TD, whilst those sized 11-13 mm on the day prior to triggering were most contributory^27^. The aforementioned studies employed ML methods which show predictor importance measures against the desired outcome (oocytes retrieved), and therefore provide a useful data-driven target for oocyte maturation based upon many previous IVF cycles^25–27^. Transparent models such as these should be favored at embryonic stages of AI-driven developments, to ensure clinicians and patients can gain trust towards CDSSs as part of ART workflows^28,29^. It is crucial to take into account the nuances of workload management in daily clinical practice in order to incorporate AI models into workflows effectively^30^. Real-world data where ultrasound scans may not be conducted every day can challenge the precision of models developed to assess TD or misrepresent the predictive capacity of certain features.

A proof-of-concept CDSS by Letterie and MacDonald (Table 2) also considered a decision point to trigger or cancel the cycle^30^. This notion was further developed in a later study looking specifically at TD assignment to optimize the retrieval of oocytes^31^. Features included pre-cycle characteristics, as well as estradiol level and follicle diameters determined on the single ‘best day’ for assessing TD, for which baseline AMH alone was most predictive^31^. A stacking model was trained, which compounds the predictive power of multiple ML models to improve overall robustness. This CDSS fulfills the need for streamlining follicular monitoring that may arise from reasons such as long-distance travel to clinics or unprecedented public health constraints. In response to the constraints enforced by COVID-19, Roberston et al. demonstrated that day-5 of OS would be the ‘best day’ for predicting both the risk of OHSS and optimal TD^32^. Both these studies highlight reducing monitoring in certain clinical settings may be possible, which could reduce resource requirements in the clinic, and the burden upon patients. The timing of the TD is a multifaceted decision point and therefore to confirm utility in practice, prospective validation of the developed models in diverse populations would be a prudent next step forward.

## In the embryology laboratory

The application of AI in the embryology lab has attracted significant recognition in recent years and has been reviewed comprehensively^33,34^, with more recent developments summarized here (Tables 4, 5, and 6). The capacity of AI techniques to analyze large amounts of complex data such as images and time-lapse objectively, whereby non-invasive assessment of gametes and embryos can be done in real-time, has significant potential for future impact in achieving healthy live birth. This can lessen the need for specialist embryology resources whilst automating some of the processes involved to reduce costs.

**Table 4:** Sperm assessment studies using artificial intelligence

Study	ART process	Outcomes of interest	Dataset	AI methods	Results
Hicks et al. (2019)^45^	Motility assessment	-Sperm video sequences used to predict motility in terms of progressive, non-progressive, and immotile spermatozoa. -Combined with participant data in multimodal analysis for automated prediction of motility parameters.	VISEM—live spermatozoa videos from 85 different participants.	Deep learning—CNN	-Deep learning algorithms capable of predicting sperm motility efficiently and with reproducibility. -Combination with participant clinical information did not improve prediction. -Incorporation of temporal analysis outperformed traditional machine learning approach. -Best MAE achieved with CNN was 8.74.
Thambawita et al. (2019)^46^	Motility assessment	-Extraction of temporal features from sequential frames from videos are able to predict motility and train traditional CNN models.	VISEM	Deep learning—CNN	-Increase in number of stacked video frames from 9 to 18 improves motility prediction, implying this model has capabilities to learn temporal features from different video frames. -Best MAE achieved with CNN was 8.74.
Ottl et al. (2022)^47^	Motility assessment	-Automatic sperm motility assessment using framework of unsupervised spermatozoa tracking, feature extraction, and ML.	VISEM	Linear Support Vector Regression	-Able to predict the percentage of progressive, non-progressive, and immotile spermatozoa in a given sample. -MAE reduced to 7.31; an improvement compared to previous papers.
Saiffe Farías et al. (2022)^48^*	Motility assessment	-Individual operator-assessed single sperm morphology linked to motility patterns assessed by vision-based AI software in ICSI ready sperm.	2154 individual sperm video recordings.	Vision-based AI Software SiD1 (IVF2.0 Ltd.)	-Spermatozoa classified as morphologically normal showed better motility variables (higher linear movement, straight line velocity). -Sperm tail morphology defects had the most significant impact on motility variables. -AI-driven sperm motility assessment may be sufficient to assess morphological features for sperm selection.
Mendizabal-Ruiz et al. (2022)^49^	Motility assessment	-Vision-based AI software assessing progressive motility parameters (straight-line velocity, linearity of curvilinear path, head movement patterns) to predict successful fertilization and blastocyst formation.	383 individual spermatozoa videos from 78 ICSI cycles.	Vision-based AI Software SiD1 (IVF2.0 Ltd.)	-Statistically significant differences in progressive motility patterns measured by SiD1 between successful and unsuccessful fertilization, and blastocyst formation. -Possible avenue for carrying out real-time analysis of individual spermatozoa during selection for ICSI.
Shaker et al. (2017)^52^	Morphology assessment	-Sperm images labeled with a class were divided into patches to identify important features in the sperm. -Dictionary learning is more effective for sperm head classification than previously published shape-based feature recognition. -Developed HuSHeM dataset with consensus classification and freely available for research purposes.	HuSHeM—includes 216 sperm head images (54 normal, 53 tapered, 57 pyriform, and 52 amorphous). SCIAN-MorphoSpermGS—includes 1862 images of sperm shapes (100 normal, 228 tapered, 76 pyriform, 73 small, and 656 amorphous), partial consensus among three experts.	Traditional ML with adaptive patch dictionary learning	-62% accuracy with SCIAN-MorphoSpermGS dataset. -92.3% accuracy, 93.5% precision, and 92.3% recall with new HuSHeM dataset.
Javadi and Mirroshandel (2019)^56^	Morphology assessment	-Deep CNN trained to detect morphological deformities in head, acrosome, and vacuole. -Developed MHSMA dataset labeled with normal sperm (acrosome, head, vacuole, tail, and neck).	MHSMA—includes 1,540 sperm images from 235 subjects with male factor infertility.	Deep learning—CNN	-High accuracy for detection of morphological deformities in sperm acrosome, head, and vacuole. -Accuracy scores of 76.7%, 77%, and 91.3% in acrosome, head and vacuole abnormality respectively, which requires improvement. -Able to classify images in real-time, aiding in selection of sperm for ICSI.
Abbasi et al. (2021)^57^	Morphology assessment	-Deep CNN algorithms trained to detect morphological deformities in head, acrosome, and vacuole.	MHSMA	Deep learning—CNN	-AI models capable of predicting sperm head features more accurately than previous study (84%, 80.7%, and 94% for sperm head, acrosome, and vacuole respectively).
Jiang et al. (2022)^58^*	Morphology and viability assessment	-Deep learning AI technique to predict viability of immotile sperm through morphology assessment with a single bright-field image.	1471 images of immotile sperm from 15 semen samples for training 10 new semen samples for validation.	Deep learning—CNN	-AI model able to accurately predict sperm viability in non-invasive manner without sample processing or staining. -Subtle morphological changes to sperm nucleus detected by AI otherwise challenging to identify with the naked eye. -Yet to be externally validated.
Joshi et al. (2023)^124^	Morphology assessment	-Deep neural network for morphological classification of sperm sample videos captured at 40x objective magnification.	-32 cryopreserved donor semen samples with known teratozoospermia and 720 vitrified sibling-oocytes from donors. -Oocytes split evenly between two conditions: (1) standard ICSI performed according to laboratory protocols and (2) AI-assisted sperm selection prior to injection.	Deep learning—CNN	-AI-ICSI group resulted in relatively increased fertilization by 6.42% and blastocyst rate by 21.35%. -Formation of high quality blastocysts increased by 41.7% compared to standard embryologist selection.
McCallum et al. (2019)^62^	DNA fragmentation	-Correlation between spermatozoa image and DNA integrity from single bright-field image.	1064 images of stained sperm with known DNA integrity.	Deep learning—CNN	-Deep CNN trained to predict DNA integrity from single spermatozoa image in under 10 ms.
Kuroda et al. (2022)^63^*	DNA fragmentation	-Modified AI-aided sperm chromatin dispersion (SCD) counting device compared to conventional Halosperm G2 Test.	17 semen samples	AI-driven SCD Kit	-AI-aided automatic counting device capable of determining DNA fragmentation quicker in a much larger sample (mean 500 spermatozoa analyzed manually in 20 min. vs. 2600 spermatozoa analyzed automatically in 5 min.), and with good correlation to conventional testing. -Automated AI device ‘X12’ had good correlation to conventional Halosperm G2 test (*r* = 0.69, *p* = 0.02), as well as the group’s modified SCD R10 manual test (*r* = 0.88, *p* < 0.01).
Peng et al. (2023)^64^	DNA fragmentation index	-ML-based clustering used to determine the effect of DNA fragmentation index and conventional semen analysis parameters on IVF outcomes.	1258 fresh IVF cycles with DNA fragmentation index data.	Unsupervised *k-*means clustering	-Favorable IVF outcomes seen with low sperm DNA fragmentation values, in combination with high or moderate motility sperm concentration and motility levels. -Worst outcomes seen with high sperm DNA fragmentation values and low sperm motility and concentration levels (live birth odds ratio 0.62; 95% CI 0.39–0.97).

Summary of studies using artificial intelligence (AI) and machine learning (ML) methods for sperm assessment and selection. The asterisk (*) indicates studies from conference proceedings. *MAE* mean absolute error, *CNN* convolutional neural network, *ICSI* intracytoplasmic sperm injection.

**Table 5:** Oocyte assessment studies using artificial intelligence

Study	Outcomes of interest	Dataset	AI methods	Results
Kanakasabapathy et al. (2020)^72^*	Whether the addition of synthetic oocyte images generated by a pretrained GAN would improve the performance of a CNN in oocyte assessment.	-Synthetic CNN trained using 1411 oocyte images and 1340 synthetic oocyte images generated by a GAN.	Deep learning—CNN and synthetic GAN.	Synthetic oocyte images generated by a pretrained GAN was able to help a CNN outperform conventionally trained CNN to determine oocytes that fertilized normally or abnormally (67.0 vs 82.6% accuracy).
Nayot et al. (2020)^74^*	CNN based visual assessment tool to predict fertilization and blastocyst development compared to expert embryologists.	CNN based on 17,659 2D oocyte images. Validation studies consisting of balanced 300 oocyte images (100 failed fertilization, 100 fertilized but did not reach blastocyst stage, 100 that reached blastocyst stage).	VIOLET™ (Future Fertility) deep learning AI image analysis tool (CNN).	-Violet outperformed 17 embryologists from 8 IVF clinics to accurately predict fertilization (71.7% vs 58.9%) and blastocyst development (62.8% vs 52.2%). -Reproducible results in a second validation study. -AI outperforms manual assessment in oocyte morphology assessment.
Mercuri et al. (2022)^75^*	Oocyte images analyzed and scored by image analysis AI tool predicting quality of blastocyst development.	16261 oocyte images from 5620 subjects with known clinical outcomes	MAGENTA™ (Future Fertility) AI image analysis tool.	-Magenta tool score correlated with blastocyst quality in stepwise manner. -Tool was able to differentiate between non-blastocyst and low quality blastocyst (ICM or TE grade of C or D) as well as low quality blastocyst and medium/high quality blastocyst (ICM and TE grade of A or B).
Link et al. (2022)^76^*	Prediction of oocyte developmental potential to top quality day-5 blastocyst from cumulus oophorus cells compared to expert embryologist.	65 cumulus cell samples from oocytes of 26 patients.	8 ML models and 25-gene network—OsteraTest bioinformatics tool.	-Cumulus cells from oocytes underwent real-time PCR with 25 target genes. Gene expression levels are computed by ML models to indicate developmental potential of each oocyte. -86% accuracy in predicting oocyte developmental capacity into a top quality blastocyst. -Yet to undergo a large-scale, prospective, randomized study for external validation.

Summary of studies using artificial intelligence (AI) and machine learning (ML) methods for oocyte assessment, prediction, and selection. The asterisk (*) indicates studies from conference proceedings. *CNN* convolutional neural network, *GAN* generative adversarial network, *ICM* inner cell mass, *TE* trophectoderm, *PCR* polymerase chain reaction, *AUC* area under curve.

**Table 6:** Embryo assessment studies using artificial intelligence

Study	ART process	Outcomes of interest	Dataset	AI methods	Results
Khosravi et al. (2019)^85^	Prediction of blastocyst quality (poor vs. good).	-Classification of blastocyst quality at 110 hrs. post insemination.	Retrospective dataset consisting of 12,001 time-lapse images at 110h post insemination.	Deep learning—CNN	-Development of AI model (STORK) to predict blastocyst quality. -Predicted blastocyst quality with AUC above 0.98. -AUC of 0.90 and 0.76 achieved on validation with two external datasets.
Dimitriadis et al. (2019)^81^	Determination of normal fertilization (2PN vs. non-2PN embryos).	-Categorization of embryos based on fertilization outcomes.	Retrospective dataset of 3469 embryos (2893 2PN; 576 non-2PN).	Deep learning—CNN	-AUC of 0.90, with PPV of 96.2% and NPV of 78.1%. -Trained CNN capable of automated fertilization check with high accuracy.
Fukunaga et al. (2020)^82^	Pronuclei determination.	-Categorization of oocytes based on pronuclei status.	Retrospective dataset of 900 embryos (300 each 0PN, 1PN, and 2PN).	Deep learning—CNN	-Precision of machine learning equivalent to that of expert embryologist. -Sensitivity for detection of 0PN, 1PN, and 2PN: 99%, 82%, and 99%, respectively.
Coticchio et al. (2021)^83^	Cytoplasmic movement to predict blastocyst development.	-Deep learning methods based on cytoplasmic movements at early cleavage stage to predict development to blastocyst.	Retrospective analysis of 230 embryo time-lapse sequential images.	Deep learning ANN extended by *k*-NN.	-Combination of blind operator assessment and deep learning models led to prediction accuracy of 82.6%, 79.4% sensitivity and 85.7% specificity. -Highlights importance of cytoplasm dynamics as novel source of data.
Zhao et al. (2021)^84^	Labeling of segmented day-1 embryos.	-CNN labeling of zona pellucida, cytoplasm, and pronuclei performance compared with manual labeling by a clinical embryologist.	1218 images from 24 day-one embryos of 14 subjects.	Deep learning—CNN	-Good precision in measurement of cytoplasm, pronuclei, and zona pellucida (97%, 84%, and 80% accuracy respectively) and comparable with morphometrics reported in literature. -Rapid labeling of all images: 130 hrs. for manual labeling against 12.18 s for CNN.
Thirumalaraju et al. (2021)^86^	Blastocyst classification based on morphological data.	-Classifying blastocysts based on morphological data in eight different neural network architectures.	742 embryo images used for validation.	Deep learning—CNN	-XCeption CNN architecture correctly classified > 99.5% of the highest quality blastocysts as good embryos. -Accuracy of Xception model in categorizing blastocyst and non-blastocyst was 90.9%.
Berntsen et al. (2022)^87^	Embryo selection for transfer.	-Prediction of implantation outcome with fully automated deep learning tool.	115,832 embryo time-lapse sequences (validation set of 17,249 embryos, 2212 with known outcomes).	Deep learning—CNN (iDAScore v1).	-AUC of 0.95 in predicting implantation when all embryos are considered together (including 1510 embryos labeled as discarded due to manual deselection by embryologist or aneuploidy). -Inclusion of discarded embryos in model training aids deep learning.
Hickman et al. (2022)^95^*	Embryo selection for transfer.	-CHLOE EQ™ score based on embryo bioinformatics and relation to expert embryologist grading, implantation, and live birth.	799 day-5 embryo time-lapse videos	Not disclosed	-CHLOE EQ™ score was directly related to embryologist ranking of morphology. -CHLOE EQ™ score differentiated between embryos that implanted and those that did not. -Strong correlation between human and AI-determined morphokinetic labeling. -Was not predictive of live birth.
Diakiw et al. (2023)^89^	Embryo selection for transfer.	-AI model using deep CNN and Grad-CAM++ mapping.	-9359 day-5 blastocyst images from 4709 women who underwent IVF.	Deep learning—CNN	-Heat maps generated for regions relating to viable and nonviable embryo classification and AI score generated. -Positive linear correlation of AI scores with pregnancy outcomes were found, leading to 12.2% reduction in time to pregnancy in comparison with standard morphological grading methods. -AI scores significantly correlated with Gardner morphological score and associated with embryo ploidy status.
Meseguer Escriva et al. (2022)^99^*	Aneuploidy assessment	-AI model using 5 feature extraction models to predict ploidy status (abnormal morphokinetic patterns, an embryo grading classification algorithm, differential cell division activity, mitochondrial DNA content, and quantification of blastocoelic contractions).	Retrospective dataset of 2502 embryo time lapse sequences with known ploidy status.	Deep learning—CNN	-Integration of all 5 features led to 90% accuracy in prediction of ploidy status. -Non-invasive AI-guided PGT triage could be a useful adjunct to conventional embryo selection or recommendation for PGT.
Barnes et al. (2023)^100^	Aneuploidy assessment	-Prediction of ploidy status based on static images, morphokinetic parameters, morphological assessments, and maternal age.	Retrospective dataset of 10,378 annotated blastocysts from 1385 patients with known ploidy status.	Deep learning—CNN	-‘STORK-A’ automated embryo evaluation predicted aneuploid versus euploid embryos with an accuracy of 69.3% (AUC 0.761) when using images, maternal age, morphokinetics, and blastocyst score. -Accuracy increased to 77.6% in prediction of complex aneuploidy vs. euploidy. -Two external test datasets, achieved an accuracy of 63.4% and 65.7%, showing generalizability.

Summary of studies using artificial intelligence (AI) and machine learning (ML) methods for embryo assessment, prediction, and selection. The asterisk (*) indicates studies from conference proceedings. *PN* pronuclear, *AUC* area under curve, *CNN* convolutional neural network, *ANN* artificial neural network, *k-NN* *k*-nearest neighbor.

## Sperm assessment

### Computer-aided sperm analysis

Standard semen analysis comprising of concentration, motility, and morphology assessment remains the first-line investigation of pre-treatment male fertility potential. Computer-aided sperm analyzers (CASA) aim to reduce intra-operator subjectivity and variability associated with manual assessment while standardizing and increasing throughput capacity. CASA analysis of sperm concentration and motility have shown a good correlation with manual assessment^35^, while estimates of progressive motility are also significantly linked to both in vivo and in vitro fertilization rates^36–39^. However, CASA-based morphological assessment tends to correlate the least with manual assessment, likely as a result of heterogeneity within a given semen sample and the subjective nature of interpretation^35^.

The latest WHO manual on sperm analysis^40^ (2021) recognized the ability of CASA to accurately determine sperm concentration and progressive motility parameters through the use of fluorescent DNA stains and tail-detection algorithms^41^. These advancements have improved the distinction between immotile spermatozoa and particulate debris; a problem that has led to the overestimation of concentration, and underestimation of progressive motility, since the inception of computer-aided systems.

At a population level, ML algorithms could be a useful to identify individuals at risk of an abnormal semen profile. An ANN based on an 11-question demographic characteristic questionnaire (including age, alcohol consumption, smoking status, urbanization and occupational exposures) achieved 92.9% accuracy in predicting abnormal sperm concentration, and 85.7% for predicting any sperm abnormality^42^. Although only developed in a small cohort of 141 men, if replicated, an AI-driven triage model could be used as a preliminary screening tool with early recourse to diagnostic testing.

Further, an ANN using semen parameters as inputs in 177 men was able to predict seminal plasma biochemical markers including fructose, zinc, and total protein content^43^. The added value of these biochemical parameters over standard semen analyses is still unclear, but a number of omics-based markers in seminal fluid have been identified as helpful in determining fertilization prognosis in a cost-effective manner^44^. Incorporating these techniques into the IVF clinic is challenging, namely due to initial set up costs and specialized techniques required for analysis. Moreover, whether these markers and profiles could drive selection of an individual spermatozoon for fertilization remains unclear.

### Motility

Accurate assessment of sperm motility is paramount in fully understanding genetic and biochemical factors that may impact normal fertilization and thus plays a key role in selection for ART. Motility prediction based on deep learning using sperm videos has been examined with promising results^45–47^. AI software may begin to allow correlation of kinetic motility patterns with other crucial factors such as sperm morphology, likelihood of fertilization, or blastocyst formation to aid in selection for intra-cytoplasmic sperm injection (ICSI) in real-time^48,49^. These studies show the potential of incorporating temporal features into deep learning models to extract insights into sperm motility consistently and efficiently.

### Morphology

Staining of spermatozoa is currently required to identify morphological abnormalities and defects for diagnostic purposes. However, given that the staining of sperm affects their vitality and motility, tested spermatozoa are no longer viable for use in ICSI and thus, do not aid in sperm selection for fertilization^50^. Consequently, morphological assessment of a single spermatozoon in a non-invasive manner using AI techniques is of interest for sperm selection^34^. Some models consider specifically the sperm head morphology^51–54^, whereas others consider a more comprehensive analysis of the whole sperm^55^.

WHO describe eleven different sperm head abnormalities by taking into account shape, size, and consistency^40^. Some of these subtypes present further challenges, with their morphology forming a vast continuum with overlaps, such that discrimination is complex to the naked eye. Using a dictionary learning approach combined with segmented microscopic sperm head images, Shaker et al. achieved a 92.3% accuracy in distinguishing between four sub-types against a ground truth dataset agreed by three experts^52^.

Open datasets of spermatozoa are becoming accessible to researchers and have been used to benchmark different models against one another^51,52,56^. Latest deep learning advancements with CNNs are capable of detecting morphological deformities in spermatozoa head, acrosome, and vacuole in real-time using low-magnification microscopes (400-600x) without staining and with increased objectivity^56,57^.

Non-invasive AI methods are also capable of assessing morphological features of immotile or frozen sperm that are difficult to characterize manually. Current viability tests require cytotoxic staining that renders individual spermatozoon unusable for ICSI. Recently, Jiang et al. described an AI model capable of identifying viable sperm based on a single bright-field image without the need for any sample processing or reagents^58^. The model exhibited 94.9% accuracy, 97.0% sensitivity, and 93.3% specificity, based on subtle morphological changes to the cell nucleus. Incorporation of such AI models into existing CASA systems could further reduce the need for sperm staining in the future, especially in the context of surgically retrieved or frozen sperm with unknown viability.

To our knowledge, no computer-aided systems exist to improve the surgical retrieval of sperm yet. Current testicular sperm extraction techniques for ICSI can be challenging, with outcomes being greatly operator-dependent^59^. However, AI techniques to aid identification of sperm from biopsies during testicular sperm extraction have been investigated. Wu et al. describe a deep CNN capable of finding sperm in testicular biopsy samples with good accuracy (mean average precision of 0.74) but did not compare this to standard embryology techniques^60^. ML models employing 16 preoperative assessment variables (e.g., hormonal parameters, genetic, demographic, lifestyle, and urogenital history) have also been shown with moderate performance to predict the success of testicular sperm extraction^61^. Given the clinical implications of not pursuing surgical sperm retrieval (i.e., unequivocal use of donor sperm), further external validation of this promising model is required. The inclusion of additional biomarkers such as more detailed genetic information, seminal plasma microRNA, or additional hormones, as a way of further improving model performance, would also be of interest.

Sperm selection for ICSI is not standardized and WHO guidelines are interpreted subjectively by embryologists. High-throughput AI models have the potential to be more objective and tackle the fundamental challenge of selecting individual sperm with the best potential for embryo formation from a sample of over 10^8^ gametes^50^. Nonetheless, with respect to morphology, there are currently no studies that assess AI performance against manual assessment according to WHO guidelines^34^. Indeed, the potential performance of AI networks is directly linked to the quality of the database used for training, as well as the caliber of data used as input. Progress on its use in sperm selection would benefit from global collaboration between clinical and laboratory teams to build a robust and definitive database of sperm images to establish a consensus ground truth.

### DNA fragmentation

Existing techniques for sperm DNA fragmentation similarly lack data at the single spermatozoon level. Modern-day tests of DNA integrity are invasive and conducted at the sample level, making them an unsuitable metric in the selection of individual sperm for ICSI. McCallum et al. described a CNN trained using a set of 1064 images of individual sperm cells of known DNA integrity to provide a DNA integrity prediction from a single bright-field image in under 10 ms^62^. Recently, Kuroda et al. described further progress with their AI-augmented sperm chromatin dispersion (SCD) test kit capable of assessing DNA fragmentation in >5000 spermatozoa at once, compared to a limited 300 in the widely commercially-used Halosperm SCD test^63^. The improved kit showed a good correlation with the conventional test that requires manual counting (Halosperm G2; *r* = 0.69, *p* = 0.02). DNA fragmentation counting took 5 min. in the automated device compared to around 20 min. with the manual method^63^.

Emerging evidence increasingly suggests that sperm DNA fragmentation is associated with reduced male reproductive capability and can be assessed in combination with conventional sperm analysis^64^. However, routine testing remains contentious and may not necessarily provide predictive value^65^. Other technical limitations exist, in particular the use of different staining, microscopes, and assays for DNA fragmentation that can challenge the training of an accurate AI model. Guidelines for testing, and optimal techniques for testing sperm DNA fragmentation have been proposed^66,67^, but testing is still not widely recommended. Progress in this field thus relies on the standardization and optimization of DNA fragmentation assays, prospective evaluation of its impact on ART outcomes, and the development of therapies to improve sperm DNA fragmentation levels^68^. Should this be achieved, ML algorithms that can combine morphological, motility, and DNA fragmentation data with outcomes such as fertilization, miscarriage, and live birth rates, could standardize, and vastly improve, single sperm assessment/selection by reducing the subjective and inter-variable outcomes between embryologists.

## Oocyte assessment

Nuclear maturity of human oocytes can only be verified by observation of the extruded polar body, which requires removal of the cumulus^10^. Automated, non-invasive methods to assess nuclear and cytoplasmic maturity and future reproductive potential would be desirable, particularly for fertility preservation. Accurate prediction of oocyte quality and fertilization prospects would allow better estimation of personalized live birth predictions from a pool of cryopreserved oocytes. Consideration of whether this is sufficient to realize a desired family size may dictate the need for further cycles of OS and cryopreservation. Clinicians would also be able to manage expectations for success and reduce the number of poor-quality embryos with low implantation potential^69^.

Currently, assessment of nuclear oocyte maturity is performed visually by embryologists in a subjective manner prior to fertilization. Oocyte scoring systems assessing cytoplasmic morphological features such as the presence of vacuoles, degree of perivitelline space, and cytoplasmic granularity, among others, have long been proposed as predictors of insemination outcome but remain points of contention as prognostic indicators of embryo development and implantation^70,71^. Substantial labeled datasets of oocytes are scarce—as such, Kanakasabapathy et al. combined a retrospective dataset of oocyte images with known fertilization outcomes alongside synthetic oocyte images generated by a GAN to form a synthetic CNN^72^. This synthetically-extended CNN outperformed the raw CNN, and delivered an accuracy of 82.58% with an AUC of 0.81 in identifying oocytes that would fertilize normally to form two-pronuclear zygotes (2PNs), versus those that would not (non-2PNs)^72^. This study showed the value of using AI to augment the training, predictive power, and robustness of existing CNNs available for the embryology lab, perhaps widening their scope of use in ART^73^.

A non-invasive CNN-based software, VIOLET™ (Future Fertility), has been shown to predict fertilization and blastulation with 91.2% and 63% accuracy respectively, based on morphological features of 2D oocyte images. The tool’s performance was much quicker and also outperformed expert embryologists in accuracy^74^. VIOLET™ aims to give users undergoing oocyte cryopreservation a personalized estimate of live birth potential based on the morphology of oocytes cryopreserved as opposed to generalized age-related outcomes. Similarly, the MAGENTA™ tool employs 2D images of denuded oocytes and a similar morphology-based CNN to score oocytes and predict the potential for high-quality blastocyst formation with good accuracy^75^. Though promising in correlating oocyte morphology with blastocyst potential, their estimates lack interplay with potential male factor subfertility and could benefit from the incorporation of clinical variables such as BMI or endometriosis, to enhance the prediction of outcomes such as clinical pregnancy or live birth.

More recently, a non-invasive gene expression test was prospectively trialed by Link et al.^76^. The ‘OsteraTest’ software is composed of eight ML modules and uses a 25-gene network to predict oocyte quality based on cumulus cells^76^. This bioinformatics-inspired approach was able to non-invasively predict oocyte development to a day-5 blastocyst with 86% accuracy^76^. Though further large-scale validation is necessary, this type of AI approach could change current practices in oocyte selection prior to cryopreservation and ICSI, as well as reduce the pool of embryos formed, cryopreserved, and tested, prior to embryo transfer. This may be particularly beneficial in countries with regulatory frameworks surrounding embryos such as Poland, where only six oocytes may be fertilized per cycle, or Germany where no more than three embryos can be stored per treatment attempt. Additionally, it may guide egg sharing or donor oocyte cycles and inform on how to distribute oocytes evenly or the total within a cohort depending on blastocyst potential.

Although these approaches provide direction for further research, the data must be viewed with caution until published in peer-reviewed journals. In developing an AI model, it is imperative to define a set end goal such as oocyte quality following oocyte cryopreservation. If fertilization is planned and blastocyst potential is being predicted, then spermatozoon quality and other male confounders should be considered. Proposed biomarkers to predict oocyte potential include follicular fluid markers (insulin-like growth factor, zinc levels^77^), cumulus-oocyte complex composition^78^, and cytoplasmic features like mitochondrial function^79^. Consideration of these methods to guide oocyte selection in the future would also require analysis into whether they are feasible in daily practice or in fact as cost-effective as fertilizing all suitable oocytes^80^.

## Embryo assessment

Embryo selection based on morphological assessment is an important predictor of success in IVF cycles but is primarily based on static visual observations at specific developmental time points. Information obtained in this manner is not only highly subjective with great inter-operator variability but also diminishes the dynamic nature of a developing embryo in culture, thus limiting its accuracy. AI-driven embryo analysis is suited to predicting developmental potential, non-invasive aneuploidy assessment, and ultimately the selection of an embryo with the best live birth potential for transfer.

### Morphokinetics and morphology

Examples of developments in embryo evaluation include the assessment of pronuclear stage embryos to differentiate between 2PN and non-2PN zygotes^81,82^. Morphokinetic data such as cytoplasmic movements have also shown potential to predict blastocyst formation at early cleavage stages in a time series-based ANN model^83^. Further assessments of interest include morphological classification of pronuclei size and arrangement to monitor embryo development^84^. CNN models showed comparable results to manual labeling, albeit with high precision and reproducibility at a fraction of the time required by clinicians (12.18 s vs. 130 hrs.)^84^. Despite promising results, the standard morphological assessment remains the international consensus which is subjective and labor-intensive.

Time-lapse images combined with automated morphology assessment of embryos based on CNNs have shown promise, capable of outperforming individual embryologists with excellent accuracy^85,86^. Other fully automated deep learning-based models using time-lapse images such as iDAScore (Vitrolife) have shown the ability to accurately assess embryo morphology without the need for concurrent embryologist assessment or annotation, and predict implantation outcome^87–89^. The benefit of using time-lapse incubation systems and/or AI technology in the embryo selection process is yet to be proven as superior to current means in double-blind RCTs^90,91^. The SelecTIMO trial recently showed no improvement in cumulative live birth rates when using uninterrupted culture conditions with routine morphological embryo selection compared to a time-lapse based embryo selection algorithm alongside uninterrupted culture for day-3 embryos^92^. With no improvement in cumulative pregnancy rates or time-to-pregnancy, it may be that the time-lapse selection method may not improve pregnancy rates, however, whether this applies to day-5 embryos is still to be clarified. Nevertheless, the time-lapse technology was not inferior and therefore could achieve similar outcomes in an automated and less subjective manner. Importantly, with modern advancements in cryopreservation, it is likely that the most viable embryos will eventually be transferred if needed. Additionally, human input may be needed to aid the assessment of embryo quality, for example, by repositioning embryos to get a better view, which should be taken into account when considering the application of an AI for this task. Validation data from the VISA Study (ClinicalTrials.gov Identifier: NCT04969822), a noninferiority, prospective, multi-center RCT may further reflect the clinical impact of AI-driven systems compared to manual morphology assessment by embryologists for day-5 embryos. Such studies highlight the necessity for the accuracy of predictions made via AI techniques to be prospectively validated prior to adoption into clinical practice with appropriate mitigation of study biases and evaluation of cost-effectiveness^20,93^.

Recently, a biomarker-scoring CDSS based on 799 blastocyst videos, CHLOE EQ^™^ (Fairtility), has been described and takes into account patient and embryo data including blastocyst diameter, degree, and time of expansion, and other morphokinetic markers. Though preliminary results are promising, these new systems still require external validation and larger-scale prospective studies before widespread adoption to realize the end goal of fully automated blastocyst assessment and accurate embryo prognosis^94,95^. It is paramount that future algorithms focus not only on the competitive selection of the best embryos for culture and transfer but also can differentiate between embryos that are otherwise morphologically indistinguishable to the naked eye, wherein the real challenge lies.

### Aneuploidy

Rates of pre-implantation genetic testing for aneuploidy (PGT-A) as a screening tool to improve clinical outcomes in ART cycles have increased in recent years. Currently, PGT-A is performed by trophectoderm biopsy on blastocysts followed by whole-genome or targeted DNA amplification and a next-generation sequencing assay. Multiple blinded non-selection studies have now shown a high prognostic failure of live birth when an aneuploid result is obtained^96,97^. Furthermore, discarding uniformly aneuploid embryos is unlikely to have a meaningful impact on cumulative live birth rates, especially in women over 35 years of age where it is more likely to be employed^98^. As modern invasive techniques still bring technical and financial challenges, non-invasive AI-driven PGT-A could offer the benefits of PGT-A without embryo manipulation and biopsy. Recent single-center studies have shown ongoing validation of AI models feeding time-lapse imaging data into CNNs to predict ploidy status from abnormal morphokinetic patterns with good accuracy^99,100^. These models may not replace PGT-A but highlight the potential for PGT-A triage and well-informed guidance towards embryo selection in a non-invasive manner^99–102^. Once again, further validation and large multi-center datasets must be compiled for standardization and generalization of these AI-driven models.

### Omics

A comprehensive understanding of the embryo at a molecular level may present another adjunct for the high throughput and comprehensive capabilities of AI-driven predictive models in the future. Various metabolomic signatures of an embryo have been investigated over the years, mainly pertaining to metabolites or biomarkers in spent culture media as a reflection of complex physiological and pathological responses and in turn, reproductive potential or ploidy status. Conflicting results to this approach have been shown^103–107^, while a previous meta-analysis including four RCTs and a total of 924 women showed no meaningful effects for metabolomic assessment on clinical outcomes^108^. Interestingly, an ANN employing a combination of conventional embryological data and thirteen nuclear magnetic resonance spectroscopy-identified metabolite levels has shown promise in predicting blastocyst implantation, though at a very small scale with a test dataset of twelve spent culture media^109^.

Current limitations of the omics approach lie within the vast variability in culture media components used and handling of spent media, contrasting infertility phenotypes, definitive biomarkers predictive of reproductive potential, and a general lack of conclusive evidence that fertility outcomes can be optimized through omics profiling. Though non-invasive, highly specific, and perhaps crucial towards a better understanding of gamete development, it is unclear whether omics profiling can effectively contribute to an improvement in clinical outcomes or will remain principally a research tool^110^. Furthermore, the complexities of omics analysis and interpretation of output data present significant barriers to adoption in daily laboratory practice.

Embryo quality aside, reproductive outcomes also depend on implantation and the endometrium. The construction of models should also integrate features of the uterus and crosstalk between an embryo and the endometrium. To date, the clinical benefit of an endometrial receptivity array (ERA) for assessment has yet to be proven^111^. The invasive nature of biopsy for endometrial receptivity testing, the time needed for results preventing immediate embryo transfer, and the potential accuracy of the diagnostic test itself are further limitations^112^. AI is however well suited to drive collaboration between ART clinics and omics-focused research groups, on account of its ability to perform large-scale data throughput and analysis. Whether these approaches will alter conventional therapies remains unclear, particularly as diagnoses such as true recurrent implantation failure and its relevance are being hotly debated currently^113^. However, given the lessons to date, the value of any ‘AI-omics’ platform should be validated in appropriately powered RCTs.

## Conclusions and future prospects

With respect to ART, several groups have developed CDSS frameworks or decision-making tools for use at key decision-points in the clinic, and/or embryology laboratory^17,30,31^. Personalization in further avenues could better improve the clinical outcomes of ART. Ovarian response has been shown to vary significantly depending on ovarian reserve, between ethnic groups^114,115^, FSH receptor genetic polymorphisms^116^, and body weight^19,117^. Therefore, incorporating such factors which influence pharmacokinetic parameters when dosing gonadotropins^9,19,20^, or suppressing premature ovulation^20,118^, may be beneficial. ML methods could also help tailor luteal phase support regimens to certain patient subgroups, where a lack of clinical consensus currently exists^119^.

The ubiquity of electronic health records (EHRs) has accelerated the development of CDSSs^15^. A predominant barrier to adoption is trustworthiness, especially with ‘black-box’ AI systems^29^, which has led to transparency being a key characteristic preferred by clinicians as such models offer simpler interpretations, although may compromise accuracy when applied to more complicated learning tasks^28^. Implementations of ‘black-box’ models are evolving, especially for embryological analyses, due to the data being primarily image-based; in turn, efforts in explainability have emerged to seek insights for model generalizability, fairness, and trustworthiness^94,95^. Misleading conclusions may be reached if clinical inference is neglected during the decision-making process since such methods are often correlation-based and prone to ‘overfitting’^120^. Generating counterfactual examples in this context, such as: “what if the optimal TD was yesterday(?)", or “what if the other embryo were implanted(?)", are generally unavailable—and to further exacerbate this—ground truths are often based upon clinical guidelines/scoring rather than objective outcome labels. The emergence of omics analyses offers an alternative, and arguably more efficient, solution for clinical and embryological assessment, although advancements currently remain of a preliminary nature^18,108^. Ultimately, appropriate assessment of CDSSs for ART is necessary in practical, ethical, and clinical contexts prior to clinical adoption. Rigorous validation with comprehensive standardized reporting is essential for establishing trustworthy models before attempting viable integration into clinical workflows^21,121^. Research conduct and reporting guidelines such as PROBAST-AI are in progress for the wider field of AI for healthcare, and with this at hand, a more granular and contextual guideline for AI in the domain of ART can be proposed^122,123^.

Salient efforts from both academia and industry have validated the utility of retrospective data to enable data-driven decision-making for ART^123^. To ensure viable deployment, these models can benefit from larger, multi-center datasets that incorporate both heterogeneous patient populations and also capture the idiosyncratic nature of clinical practice worldwide. Achieving this is best achieved through a collaborative effort from all stakeholders representing multiple disciplines across the AI and healthcare landscape^21^. Furthermore, streamlining workloads is an essential objective of CDSSs, and seamless implementation with, or within, EHR systems are essential to not inadvertently decrease the efficiency of clinical workflows. Prospective validation (e.g., well-designed RCTs) with relevant outcome measures is a key step to assess the efficacy and efficiency of these models in clinical environments and thus demonstrate impact on patient outcomes. With such efforts in place, a comprehensive end-to-end CDSS seems a plausible future goal. Whether this paradigm should extend to an autonomous AI clinician within the ART domain remains an open and contentious question. The use of AI to automate some of the tasks currently performed by clinicians or laboratory staff could have implications in training and a potential loss of expertise in the workforce, but may also free up staff time to focus on more challenging and physically demanding technical processes. Reflections on the current literature to date elicit valuable questions regarding future studies, including determining the specification of what should be measured/captured, to what precision, and how often. Decision points cannot necessarily be considered in isolation, and the relationships between some of the key topics described in this review require further interdisciplinary research to prioritize the individualization and utility of certain decisions over others. The intersection of AI and ART undoubtedly remains a nascent and valuable field of study, which has the potential to reduce intensive resources, whilst ultimately improving clinical outcomes for patients.

## Data Availability

Data sharing is not applicable to this article as no datasets were generated or analyzed during the current study.
